# Enhancing Nursing Students’ Engagement and Critical Thinking in Anatomy and Physiology Through Gamified Teaching: A Non-Equivalent Quasi-Experimental Study

**DOI:** 10.3390/nursrep15090333

**Published:** 2025-09-10

**Authors:** Sommanah Mohammed Alturaiki, Mastoura Khames Gaballah, Rabie Adel El Arab

**Affiliations:** Almoosa College of Health Sciences, Al Ahsa 36422, Saudi Arabia; s.alturaiki@almoosacollege.edu.sa (S.M.A.); m.gaballah@almoosacollege.edu.sa (M.K.G.)

**Keywords:** anatomy and physiology, critical thinking, game-based learning, gamification, nursing, nursing education, nursing students, Saudi Arabia, student engagement

## Abstract

**Background:** Gamification may enhance engagement and higher-order learning in health-care profession education, but evidence from undergraduate nursing programs—particularly in the Middle East—is limited. We evaluated whether integrating structured gamified activities into an anatomy and physiology course improves class engagement and knowledge-based critical thinking. **Methods:** In this pragmatic, nonrandomized, section-allocated quasi-experimental study at a single Saudi institution, 121 first-year female nursing students were assigned by existing cohorts to traditional instruction (control; n = 61) or instruction enhanced with gamified elements (intervention; n = 60) groups. The intervention (introduced mid-semester) comprised time-limited competitive quizzing with immediate feedback and aligned puzzle tasks. Outcomes were measured at baseline, mid-semester, and end-semester using a four-item Class Engagement Rubric (CER; scale 1–5) and a 40-item high-cognitive multiple-choice (MCQ) assessment mapped to course objectives. Analyses used paired and independent *t*-tests with effect sizes and 95% confidence intervals. **Results:** No attrition occurred. From baseline to end-semester, the intervention group had a mean CER increase of 0.59 points (95% CI, 0.42 to 0.76; *p* < 0.001)—approximately a 15% relative gain—and a mean MCQ increase of 0.30 points (95% CI, 0.18 to 0.42; *p* < 0.001), an ~8% relative gain. The control group showed no material change over the same interval. Between-group differences in change favored the intervention across CER items and for the MCQ outcome. Semester grade-point average did not differ significantly between groups (*p* = 0.055). **Conclusions:** Embedding a brief, structured gamification package within an undergraduate nursing anatomy and physiology course was associated with measurable improvements in classroom engagement and modest gains in knowledge-based critical thinking, with no detectable effect on overall semester GPA. Given the nonrandomized, single-site design, causal inference is limited. Multi-site randomized trials using validated critical-thinking instruments are warranted to confirm effectiveness and define dose, durability, and generalizability.

## 1. Introduction

Gamification—the application of game-design elements in non-game contexts—has gained traction for motivating learners and sustaining engagement, particularly in education [1,2,3]. Typical elements include points, badges, leaderboards, challenges, time limits, and immediate feedback, deployed to support motivation, participation, and problem-solving in learning tasks [1,2,3].

Recent syntheses in health and nursing education report gains in knowledge, skills, and participation with game-thinking approaches [4,5], and gamification has become increasingly prominent across educational settings, including the health sciences [6].

Within nursing education and, specifically, anatomy and physiology (A&P), applied strategies such as live quiz competitions (e.g., Kahoot), escape-room-style activities, and structured puzzle sessions have been associated with improved knowledge acquisition, engagement, and academic performance [7,8,9,10].

Cultivating critical thinking—the purposeful, systematic gathering and evaluation of information to distinguish facts from opinions, appraise evidence, and draw sound conclusions—is central to nursing education and clinical decision-making [11,12]. Evidence links gamified learning with improved learning outcomes and clinical judgment where evaluated [13], while foundational knowledge and skills relate to broader academic performance [14,15]. Clinical reasoning frameworks further anchor analytical appraisal in nursing practice [16,17], with reflection and analytical thinking recognized as core components of critical thinking [18]. In related health-care professional contexts, problem-based learning shows positive associations between problem-solving ability and critical-thinking tendency [19], and nursing-specific skill development aligns with critical-thinking disposition [20].

Educational games—including role-playing formats and smartphone applications—have been reported to support academic attainment and foster critical thinking [21,22,23]. Across settings, gamified approaches consistently raise student engagement [24,25,26], and their use in nursing education is growing with encouraging outcomes [27,28,29].

Conceptually, gamification in education is characterized by the structured use of mechanics to enhance motivation, engagement, and learning outcomes [30,31], emphasizing rewards, challenges, leaderboards, and feedback mechanisms to encourage participation and progress [32,33,34].

In the present course, gamification comprised (i) live, time-limited competitive quizzing with immediate feedback and (ii) structured puzzles (crossword, word search, hidden-message, jigsaw) aligned with weekly A&P objectives—operationalizing points/leaderboards/feedback/challenge mechanics to scaffold motivation, attention, and retrieval practice in nursing learners [2,3,7,8,9,29].

Gap and aim. Despite growing interest, evidence from Middle Eastern nursing programs and first-year A&P contexts remains limited, and many studies do not detail concrete game mechanics or course integration. We therefore evaluated the association between integrating structured gamification into A&P teaching and (a) class engagement and (b) knowledge-based critical-thinking performance, while documenting age and GPA as contextual correlates.

## 2. Method

Design and Reporting Framework. This non-equivalent, quasi-experimental study used existing class cohorts as intervention and control groups; participants were not randomly assigned. Reporting follows the TREND recommendations for nonrandomized evaluations [35] and the TIDieR checklist for describing educational interventions [36]. Baseline characteristics (age, GPA, and pre-intervention MCQ scores) were compared using independent *t* tests and chi-square tests. A priori power analysis indicated that 54 participants per group were required to detect a medium effect (Cohen’s d = 0.5) with 80% power at α = 0.05 (one-sided); no separate precision-based calculation was used.

Setting and Participants. The study was conducted in the first-year Anatomy & Physiology (A&P) course at Almoosa College of Health Sciences during a 14-week semester, in the Fall 2022–2023 academic year. In the term studied, 121 first-year nursing students were enrolled in A&P; all 121 consented and were included (Control n = 61, Gamified n = 60). Eligibility criteria were enrollment in A&P and provision of informed consent; no additional exclusion criteria were applied. Inclusion criteria were: (1) enrollment in the first-year A&P course during the study term and (2) provision of informed consent. Exclusion criteria were: (1) non-consent or withdrawal prior to baseline assessment and (2) not being enrolled on the course; no additional exclusions (e.g., repeating status or prior A&P coursework) were applied. Students were allocated to groups by existing class sections.

Participant Flow. Data were collected over three periods: TP0 (weeks 1–7), TP1 (weeks 8–11), and TP2 (weeks 12–14).

Intervention (Gamified A&P) and Comparator. Gamified sessions used jigsaw puzzles and Kahoot to teach circulatory, reproductive and lymphatic systems. Specifically, the intervention comprised (i) live, time-limited, competitive quizzing (Kahoot) with immediate feedback and (ii) structured puzzles (crossword, word search, hidden-message, jigsaw) aligned to weekly learning objectives. These instantiate points, leaderboards, challenge, and immediate feedback mechanics. Gamification was introduced at TP1 (weeks 8–11) and continued through TP2 (weeks 12–14); both sections received traditional teaching during TP0 (weeks 1–7). The control section received standard lectures, labs, and low-stakes formative quizzes without gamification. Intervention fidelity was supported by a brief delivery checklist for each session and by Kahoot participation logs.

### Outcomes and Instruments

Primary outcomes. Class engagement (CER total score) and knowledge-based critical-thinking performance (MCQ total score).

Instrument I—Sociodemographic Characteristics. Instrument I included student age and GPA. Precisely, the instrument captured age (years) and cumulative GPA on a 5-point scale (pre-semester); no other sociodemographic variables were collected. The demographic questionnaire comprised exactly two items—age (years) and cumulative GPA on the institution’s 5-point scale (pre-semester)—and was administered at baseline (TP0).

Instrument II—Class Engagement Rubric (CER). The CER comprised four items—punctuality, quality of work, critical thinking/problem solving (asking questions), and participation in class discussions—and the mean score of these items constituted the total engagement score. This rated students’ participation ability on a scale from 1 to 5, with 1 being fair and 5 being outstanding. The CER showed internal consistency (α = 0.81) and inter-rater reliability (ICC = 0.78) in pilot testing. Item content and anchors were refined for clarity following the instruction of three nursing PhD holders in the department.

Instrument III: High-Cognitive MCQ Critical-Thinking Assessment. It included ungraded quizzes (informal testing), which included 10 high-cognitive applied multiple-choice questions (MCQs), and formal exams (midterm and final), which included 30 high-cognitive MCQs. The scoring system was determined by adding one point for the correct answer and 0 points for the wrong. Items were authored by the study team and blueprint-mapped to A&P system units taught in the course (e.g., circulatory, reproductive, lymphatic). Item selection targeted the Analyze/Apply/Evaluate levels of the revised Bloom’s taxonomy and followed best-practice item-writing standards (single problem focus, plausible distractors, avoidance of cueing/“all-of-the-above”). Reliability for dichotomously scored items was estimated with KR-20 at each administration; item difficulty and point-biserial discrimination were inspected.

Pilot Study. The CER was created and tested for validity by panels of expertise and then piloted by 5% of the study sample for testing reliability (Cronbach 0.81). The pilot was conducted in week −1 (pre-semester) with a convenience subset of approximately 5% of the final cohort to assess rubric clarity and rater alignment. Pilot participants were first-year A&P students from the same intake and remained in their original sections for the main study. Minor wording clarifications were made to CER anchors; no changes were made to the intervention design or MCQ content as a result of the pilot.

Content Validation (Expert Audit). The course teacher created MCQs that would be appropriate for the nature of the course, content, included scenario, and revised by three external specialists in anatomy and physiology.

Three subject-matter experts with ≥5 years of teaching experience in nursing/health-science programs independently reviewed the CER and the MCQ blueprint and sample items for coverage, clarity, and alignment. The audit occurred before the pilot (weeks −2 to −1); feedback was reconciled by the study team. None of the experts delivered the gamified sessions or graded the formal examinations. Expert consensus indicated adequate coverage and clarity.

Procedures and Timeline. Data was collected over a 14-week semester. Time period 0 (TP0) encompassed weeks 1–7, TP1 covered weeks 8–11, and TP2 covered weeks 12–14. Both groups were taking twice a week, 120 min of theory and 120 min of lab practice. During the first period (TP0), the researcher used traditional teaching strategies in theoretical classes and lab sessions with both the control and intervention groups, also measuring students’ engagement by using the CER and critical thinking skills through MCQs, which were the results of quizzes. Topic-week mapping and activities were as follows: TP0 (Weeks 1–7)—both sections: traditional instruction; formative CER and MCQ administered; TP1 (Weeks 8–11)—intervention section: Kahoot (competitive quizzing) + puzzle sets (crossword/word-search/hidden-message/jigsaw) aligned to circulatory and reproductive systems; TP2 (Weeks 12–14)—intervention section: continued Kahoot + puzzles aligned to the lymphatic system; control section: traditional instruction only. Midterm (TP1) and final (TP2) formal MCQ exams were administered to both sections. All assessments were conducted during scheduled class sessions by the course instructor, using identical instruments and timing across groups. Kahoot analytics summarized participation and immediate feedback for the intervention section.

Statistical Analysis. Statistical analysis was performed using SPSS v20 [37]. Descriptive statistics summarized demographic characteristics. Paired *t* tests compared pre- and post-intervention scores within each group, and independent *t* tests compared change scores between groups. Effect sizes (Cohen’s d) and 95% confidence intervals were computed to quantify differences. *p* values < 0.05 were considered statistically significant; *p* values < 0.001 are reported as ‘*p* < 0.001’. Because the study used a nonrandomized design and multiple comparisons, results should be interpreted cautiously. Assumption checks were used included Shapiro–Wilk tests for normality of change scores and Levene’s test for homogeneity of variance. Where assumptions were questionable, Wilcoxon signed-rank (within-group) and Mann–Whitney U (between-group) sensitivity analyses were performed; conclusions were compared with parametric results. For Instrument III, reliability of the MCQ forms was estimated with KR-20 at each administration; Pearson correlations were used to summarize associations across occasions and with age/GPA and are reported in Results. Parametric tests were selected because assumptions were checked and met for primary contrasts.

## 3. Results

### 3.1. Participants and Flow of the Analytic Sample

A total of 121 first-year female nursing students were included in the analysis (mean age, 19.6 ± 1.4 years). Overall, 40% had an “excellent” GPA (4.51–5.00) and 26% had a “very good” GPA (3.51–4.50) at semester start. The flow of the entire analytic sample (N = 121): eligibility and inclusion; allocation by existing sections to the control group (n = 61) or the gamified-instruction group (n = 60); identification of the ≈5% pilot subset; and the timing of the intervention (introduced at TP1 and continued through TP2) and assessments (CER and MCQ at TP0–TP2). No attrition occurred.

### 3.2. Class Engagement (CER; Scale 1–5)

At baseline (TP0), item scores were similar across groups. In the control section, scores were stable from TP0 to TP2 (e.g., punctuality remained 5 ± 0.00), precluding meaningful within-group change testing for invariant items. In the gamified section, engagement improved on multiple items from TP0 to TP2: punctuality increased from 4.50 ± 0.85 to 5 ± 0.00 (*p* = 0.001), quality of work from 3.13 ± 1.07 to 4.60 ± 0.81 (*p* = 0.016), critical thinking/problem solving from 2.80 ± 1.15 to 3.10 ± 1.20 (*p* = 0.004), and participation in discussions from 2.60 ± 1.06 to 2.70 ± 1.17 (*p* < 0.001). The CER total (mean of the four items) rose from 3.26 at TP0 to 3.85 at TP2.

To standardize presentation, continuous outcomes are shown as mean ± SD with two decimals where applicable; exact integers are reported without trailing “0.00”. *p* values are reported as exact values or “*p* < 0.001” when appropriate.

Table 1 summarizes CER item scores for both sections at baseline (TP0) and end-of-semester (TP2), with corresponding tests and *p* values. Labels “Baseline (TP0)” and “End-of-semester (TP2)” replace “pre-/post-game” to avoid implying that both sections received gamification. Effect sizes (Cohen’s d) are reported in the text and, where applicable, in the table notes.

### 3.3. Knowledge-Based Critical Thinking (MCQ)

Relative to baseline, the gamified section showed a mean increase of 0.30 points on the knowledge-based MCQ assessment (95% CI, 0.18 to 0.42; *p* < 0.001), consistent with an approximate 8% relative gain. The control section showed no material change over the same interval. Between-group differences in change favored the gamified section (Table 1).

### 3.4. Semester GPA

There was no significant between-group difference in semester GPA (*p* = 0.055).

### 3.5. Assumption Checks and Sensitivity Analyses

Distributional assumptions for change scores were assessed as specified in the Methods. Normality (Shapiro–Wilk) and homogeneity of variance (Levene) checks were satisfactory for the primary contrasts. Nonparametric sensitivity analyses (Wilcoxon signed-rank, Mann–Whitney U) yielded the same inferences as the parametric tests.

### 3.6. Correlational Analyses

Table 2 summarizes associations among MCQ scores, GPA, and age. MCQ scores at TP1 and TP2 were strongly correlated (r = 0.947, *p* < 0.001), indicating temporal consistency in individual performance; associations with GPA did not reach statistical significance (*p* ≥ 0.055), and correlations with age were negligible (*p* ≥ 0.594). Table 3 presents cross-occasion correlations among formative and formal assessments (TP0–TP2). Positive coefficients between adjacent occasions (e.g., formative TP0 and formative TP1; r = 0.640, *p* < 0.001) indicate stability in rank order. Negative cross-occasion coefficients (e.g., pre-midterm vs. post-final) likely reflect shifts in content emphasis and difficulty across assessments; as simple correlations do not adjust for cohort or intervention status, they should not be interpreted as evidence of a causal intervention effect.

## 4. Discussion

In this nonrandomized, section-allocated evaluation of first-year nursing students, embedding a brief, structured gamification package—time-limited live quizzing and aligned puzzle tasks—into an anatomy and physiology course was associated with clear gains in observed classroom engagement and small improvements on a knowledge-based multiple-choice assessment, without a detectable difference in semester GPA. The gradient of effects is internally coherent—the intervention targeted three organ systems and operated within a single course, whereas GPA aggregates performance across all concurrent courses.

Several mechanisms plausibly account for the pattern observed. Live, competitive quizzing provides frequent retrieval with immediate feedback, bounded challenge, and salient progress cues; these features are known to increase attention, time-on-task, and metacognitive monitoring. Puzzle-based activities (crossword, word-search, hidden-message, jigsaw) promote elaboration of terminology and relationships among structures, supporting consolidation and near-transfer within the taught units. These mechanism profiles align with contemporary syntheses in nursing education and serious-games research showing the most consistent benefits on knowledge outcomes and learner engagement [28].

Our findings are consonant with studies that used the same game tools in undergraduate nursing and other health-care cohorts. Classroom and skills-lab implementations of Kahoot have reported higher knowledge scores, stronger motivation, and favorable learner perceptions relative to traditional sessions; examples include gains during vaccination teaching, intramuscular-injection training, arterial blood-gas interpretation, and mixed didactic contexts. Previous work focused on nursing students likewise conclude that game-thinking or digital serious games improve academic achievement, particularly knowledge. These reports support the credibility of the engagement improvements seen here and the modest, localized rise in applied MCQ performance [4,7,28,38,39].

Results also align with evidence on puzzle-based approaches for nursing learners. A randomized study in 2025 found that puzzle teaching improved knowledge, motivation, and academic self-efficacy among nursing students [40]. These convergent findings are consistent with our observed improvements in engagement and small gains in applied knowledge.

With respect to A&P, the direction of effects is also consistent with the emerging literature relevant to nursing education. Gamified question banks and e-quizzes in foundational anatomy have been associated with higher participation and short-term performance; anatomy-themed escape rooms—studied directly in nursing cohorts—have improved knowledge retention and generated strong engagement and perceived value [41].

The modest magnitude of knowledge gains and the absence of a GPA difference are readily explained. First, the dose was circumscribed (introduced after seven weeks and confined to three systems), limiting exposure relative to the traditional instruction both sections had already received. Second, the knowledge outcome emphasized applied, course-specific MCQs (near-transfer) rather than a domain-general critical-thinking instrument; larger effects on far-transfer measures would be improbable without greater intensity and duration. Third, GPA dilutes any single-course signal. Finally, allocation by existing sections permits residual confounding (e.g., study habits, peer effects, or instructor–learner dynamics), warranting cautious interpretation of between-section contrasts.

The correlational analyses are descriptive. Positive cross-occasion correlations indicate rank-order stability; negative coefficients across distal occasions likely reflect differences in content coverage and difficulty over time rather than deterioration. Because these coefficients are unadjusted for cohort or intervention status, they should not be interpreted as causal effects.

## 5. Limitations

This study has limitations. First, the course-embedded MCQ assessment—designed to map high-cognitive objectives in anatomy and physiology—has not been externally validated as a domain-general measure of critical thinking; future trials should pair course-specific MCQs with standardized critical-thinking instruments to enable construct triangulation. Second, MCQ formats primarily capture application and analysis and may insufficiently index higher-order synthesis and evaluation. Third, the non-equivalent, section-allocated quasi-experimental design precludes randomization and leaves room for residual confounding from unmeasured baseline differences. Fourth, the single-site, single-course sample of first-year female nursing students limits generalizability to other programs, academic levels, and mixed-gender cohorts. Fifth, although the Class Engagement Rubric showed acceptable internal consistency in pilot testing, external validity and rater calibration beyond this setting remain unproven. Finally, multiple comparisons were performed without formal adjustment; while exact *p* values and effect sizes are reported, future studies should prespecify primary endpoints, control familywise error, and evaluate longer-term outcomes (e.g., GPA and licensure performance). Notwithstanding these constraints, the findings support further multi-site randomized evaluations using validated outcome measures and richer assessments of higher-order reasoning.

## 6. Recommendations and Implications

The findings of this study suggest that incorporating well-designed gamification elements into anatomy and physiology curricula can meaningfully enhance student engagement and knowledge-based performance. Educators should therefore consider integrating targeted game-based activities such as interactive puzzles, case-based quizzes, and live polling into traditional lectures to stimulate active learning, foster sustained attention, and reinforce complex physiological concepts. To maximize impact, these activities must be aligned with clear learning objectives, underpinned by robust instructional design principles, and accompanied by timely feedback that guides students toward deeper understanding.

At the institutional level, nursing schools and health-sciences faculties should invest in faculty development programs that train instructors in effective gamification pedagogies and the use of digital platforms. This includes equipping educators with skills to create adaptable game scenarios that accommodate diverse learning styles and to leverage analytics from gamified assessments to identify individual students’ strengths and gaps. By embedding gamification within broader curricular frameworks rather than as one-off interventions colleges can cultivate a learning culture that actively promotes motivation, self-directed study, and peer collaboration.

For future research and policy, rigorous randomized controlled trials are needed to establish causal effects of gamification on higher-order outcomes, including true critical-thinking abilities, clinical decision-making, and long-term academic achievement such as GPA or licensure exam performance. Such trials should employ validated, domain-specific instruments (e.g., the California Critical Thinking Skills Test) and mixed-methods evaluations to capture both quantitative gains and qualitative improvements in student confidence and problem-solving behaviors. Collectively, these efforts will enable nursing education to harness the full pedagogical potential of gamification, ultimately contributing to the preparation of health-care professionals who are highly engaged, analytically adept, and ready to meet the demands of modern clinical practice.

### Ethical Considerations

This study was approved by the Research Center at Almoosa Specialist Hospital (Ethics Committee/IRB) (Approval Code: ARC-22.04.06; Approval Date: 11 May 2022). All participants provided written informed consent prior to enrollment. The study was conducted in accordance with the Declaration of Helsinki, applicable institutional policies, and local regulations. Identifiable information was not collected in study instruments; data were de-identified; stored on secure, access-restricted systems; and available only to authorized study personnel.

## 7. Conclusions

In this section-allocated, nonrandomized study of first-year nursing students, integrating a structured gamification package into an anatomy and physiology course was associated with higher classroom engagement and modest gains on a course-embedded, knowledge-based assessment; semester GPA did not differ between sections. These results, derived from a single site and a female cohort using a course-specific MCQ measure, warrant cautious interpretation. Given the nonrandomized, section-allocated design, these findings are associative and subject to confounding; multi-site randomized trials using validated critical-thinking instruments are needed to establish causal effects and durability.

## Figures and Tables

**Table 1:** Outcomes at baseline (TP0) and end-of-semester (TP2) in control and intervention groups, with within-group tests and between-group change (ΔΔ).

Engagement Item	Control—Baseline (TP0)	Control—End-of-Semester (TP2)	Control—df	Control—*p*	Intervention—Baseline (TP0)	Intervention—End-of-Semester (TP2)	Between-Group ΔΔ
Punctual on time to class, exhibits self-discipline	5 ± 0.00	5 ± 0.00	NA	NA	4.5 ± 0.85	5 ± 0.00	0.50
The student works hard and produces high-quality work.	2.8 ± 1.1	2.8 ± 1.18	32	*p* = 0.699	3.13 ± 1.07	4.6 ± 0.81	1.47
Critical Thinking and Problem-Solving Skills (asking questions)	2.2 ± 1.11	2.09 ± 1.15	32	*p* = 0.350	2.8 ± 1.15	3.1 ± 1.2	0.41
Participation in class discussions, Group team	2.6 ± 1.2	2.6 ± 1.2	32	*p* = 0.822	2.6 ± 1.06	2.7 ± 1.17	0.10
Total Average	3.15	3.12	NA	NA	3.26	3.85	0.62

NA: Not Applicable.

**Table 2:** Associations of knowledge-based critical-thinking performance with GPA and age (Pearson correlations, two-tailed).

	1	2	3	4
1. Formal test (TP1, mid-semester)	-			
2. Formal test (TP2, end-semester)	0.947 ** (*p* < 0.001)	-		
3. GPA (Grade Point Average)	0.548 (*p* = 0.055)	0.345 (*p* = 0.086)	-	
4. Age	0.042 (*p* = 0.645)	0.023 (*p* = 0.806)	0.049 (*p* = 0.594)	-

Variables: 1. Formal test (TP1, mid-semester). 2. Formal test (TP2, end-semester). 3. GPA (Grade Point Average). 4. Age. Note. ** *p* < 0.01 ** indicated with **. Report *p* as “*p* < 0.001” when applicable. All tests two-tailed.

**Table 3:** Cross-occasion correlations among informal (formative) and formal assessments (TP0–TP2).

	1	2	3	4	5	6
1. Informal assessment (TP0, baseline)	-					
2. Informal assessment (TP1, post-game)	0.640 ** (*p* < 0.001)	-				
3. Informal assessment (TP1, pre)	0.893 ** (*p* < 0.001)	0.691 ** (*p* < 0.001)	-			
4. Formal midterm exam (TP1)	0.395 ** (*p* = 0.004)	0.861 ** (*p* < 0.001)	0.404 ** (*p* = 0.003)	-		
5. Informal assessment (TP2, end-semester)	−0.250 (*p* = 0.094)	−0.704 ** (*p* < 0.001)	−0.255 (*p* = 0.087)	−0.591 ** (*p* < 0.001)	-	
6. Formal final exam (TP2)	0.563 ** (*p* < 0.001)	0.883 ** (*p* < 0.001)	0.575 ** (*p* < 0.001)	−0.760 ** (*p* < 0.001)	0.947 ** (*p* < 0.001)	-

Variables: 1. Informal assessment (TP0, baseline). 2. Informal assessment (TP1, post-game). 3. Informal assessment (TP1, pre). 4. Formal midterm exam (TP1). 5. Informal assessment (TP2, end-semester). 6. Formal final exam (TP2). Note. ** *p* < 0.01 ** indicated with **. Report *p* as “*p* < 0.001” when applicable. Two-tailed tests.

## Data Availability

The data underlying this article contain student academic records and are not publicly available to protect participant privacy. De-identified datasets may be made available from the corresponding author on reasonable request and with institutional permission.

## Public Involvement Statement

No public involvement in any aspect of this research.

## Guidelines and Standards Statement

This manuscript was drafted against the Transparent Reporting of Evaluations with Nonrandomized Designs (TREND) statement and the Template for Intervention Description and Replication (TIDieR) checklist.

## Abbreviations

A&P	Anatomy and Physiology (course context)
CER	Class Engagement Rubric (4 items: punctuality, quality of work, critical thinking/problem solving, participation)
MCQ(s)	Multiple-Choice Question(s) (high-cognitive; Analyze/Apply/Evaluate levels).
GPA	Grade Point Average (5-point scale)
TP0/TP1/TP2	Study time periods across the semester: TP0 = Weeks 1–7, TP1 = Weeks 8–11, TP2 = Weeks 12–14
TREND	Transparent Reporting of Evaluations with Nonrandomized Designs (reporting guideline)
TIDieR	Template for Intervention Description and Replication (intervention description checklist)
SPSS	Statistical Package for the Social Sciences (statistical software)
CI	Confidence Interval (e.g., 95% CI)
SD	Standard Deviation
df	Degrees of Freedom (for *t*-tests)
*p*	*p* value (two-tailed unless stated)
*d*	Cohen’s *d* (standardized mean difference/effect size)
*r*	Pearson correlation coefficient
ICC	Intraclass Correlation Coefficient (inter-rater reliability)
α	Alpha: significance level (e.g., α = 0.05); also Cronbach’s alpha for internal consistency (e.g., α = 0.81)
*n*, *N*	Sample size: *n* = subgroup size; *N* = total sample size
